# Morphological changes in alveolar bone thickness and height after orthodontic proclination or labial movement combined with autogenous soft tissue grafting: a CBCT evaluation

**DOI:** 10.1186/s12903-023-02944-w

**Published:** 2023-04-15

**Authors:** Tianyu Zhang, Lingling Zhang, Min Li, Fang Yi, Chengri Li, Yanqin Lu

**Affiliations:** 1 Department of Orthodontics, Hunan Key Laboratory of Oral Health Research & Hunan Clinical Research Center of Oral Major Diseases and Oral Health & Xiangya Stomatological Hospital & Xiangya School of Stomatology, Central South University, Changsha, 410008 Hunan China; 2 Department of Periodontics, Hunan Key Laboratory of Oral Health Research & Hunan Clinical Research Center of Oral Major Diseases and Oral Health & Xiangya Stomatological Hospital & Xiangya School of Stomatology, Central South University, Changsha, 410008 Hunan China

**Keywords:** Mucogingival surgery, Autogenous soft tissue grafting, Bone regeneration, Orthodontics, Gingival recession, Periodontal regeneration

## Abstract

**Background:**

Autogenous soft tissue grafting is indicated in thin gingival biotypes before orthodontic proclination or labial movements to increase the keratinized gingiva and prevent gingival recession. However, its effect on local alveolar bone remodeling is unclear. The aim of this study was to investigate the effects of autogenous soft tissue grafting on local alveolar bone after orthodontic proclination or labial movements.

**Methods:**

Sixteen patients with a thin scalloped gingival biotype, narrow keratinized gingiva, or thin cortical bone requiring orthodontic proclination or labial movement of teeth were included. Cone-beam computed tomography (CBCT) images were obtained before grafting and at least 6 months after surgery. Sixty mandibular teeth were included, and the vertical bone level and horizontal labial bone thickness were measured. The results were compared using paired t-tests or Wilcoxon signed-rank test.

**Results:**

The horizontal labial bone thickness increased, especially at 6 mm below the cementoenamel junction (CEJ) in the mandibular central and lateral incisors (*P* < 0.05). The total alveolar bone area of the canines, first premolars, and second premolars increased at 3, 6, and 9 mm below the CEJ, respectively, and the differences were statistically significant (*P* < 0.05). Additionally, vertical bone height increased minimally on the labial side, but the differences were not statistically significant (*P* > 0.05).

**Conclusions:**

New bone regeneration was observed on the labial (pressure) side after autogenous soft tissue grafting, which may represent a mechanism to effectively prevent gingival recession and maintain periodontal health.

**IRB approval:**

All the experimental procedures involving humans in this study were approved by the Medical Ethics Committee of Xiangya Stomatological Hospital, Central South University ( No. 20190048).

**Supplementary Information:**

The online version contains supplementary material available at 10.1186/s12903-023-02944-w.

## Background

Orthodontic mechanical forces produce tooth movement through alveolar bone remodeling. Regardless of the direction in which the tooth moves, the root should be located at the center of the alveolar bone. Adequate bone support is important for maintaining long-term periodontal stability. However, excessive movement beyond the bony support in patients with thin gingival tissue may lead to periodontal complications, including gingival recession, bone dehiscence, and fenestration [1]. Gingival recession can cause root exposure, which aggravates dentin sensitivity, and bone defects may affect the treatment stability. Therefore, to prevent gingival recession, autogenous soft-tissue grafting before orthodontic treatment has been suggested, especially in patients with thin gingival biotypes [2–4].

Autogenous soft tissue grafting is a surgical procedure that includes pedicle, free gingival, and subepithelial connective tissue grafts. Grafting is widely used to increase the volume of the keratinized gingiva. Previous studies have shown significant augmentation of the keratinized gingiva after surgery, which may prevent gingival recession [5]. However, no study has mentioned changes in the local alveolar bone. Additionally, research on whether grafting prevents periodontal recession by reducing the inflammatory response or promoting local osteogenesis is still lacking. Minimal new bone formation parallel to the root surface after soft tissue grafting has been reported in few cases [6–10]. However, there is a lack of case series that have verified the occurrence of osteogenesis, and the osteogenic mechanism remains unclear.

In patients with thin gingival biotypes, even minimal bony changes may cause or aggravate periodontal recession. Most previous studies used traditional two-dimensional images (panoramic or periapical views) to evaluate alveolar bone morphology, but the overlapping of structures inevitably caused discrepancies in assessing bone changes in three dimensions. [11] Due to technological limitations, few studies have statistically assessed morphological changes in the alveolar bone after autogenous soft tissue grafting. Therefore, studies using high-precision methods to evaluate alveolar bone conditions before and after surgery are required. Currently, CBCT has been proven to precisely depict three-dimensional bony anatomy [12, 13]. Moreover, since alveolar bone measurements using CBCT are accurate and reproducible [14], it is a reliable technology for research. Therefore, this study evaluated the horizontal labial bone thickness and vertical bone levels after orthodontic proclination or labial movement of teeth combined with periodontal grafting surgery using CBCT, with the aim of adequately assessing bony changes that could not be assessed using conventional radiography. This study aimed to explore the feasibility of autologous gingival grafting for the prevention of periodontal recession during orthodontic proclination or labial movement of teeth from the perspective of small changes in bone mass.

## Methods

### Participants

This study included 16 adult patients (4 men, 12 women; mean age 25.26 ± 7.24 years) who underwent orthodontic treatment with autogenous soft tissue grafting at our institution. The selection period was from July 2016 to December 2018.

Periodontal surgery was recommended for patients assessed to have a high risk of bone dehiscence and periodontal bone loss during orthodontic treatment by periodontologists, based on the following criteria: (1) possibility of the root moving out of the alveolar bone housing, resulting in bone dehiscence due to facial or labial movement; (2) width of the keratinized gingiva < 2 mm with width of the attached gingiva < 1 mm; (3) thin scalloped gingival biotype assessed by visual and periodontal probing [15]; and (4) alveolar bone thickness < 1 mm as evaluated using CBCT.

The exclusion criteria were as follows: (1) uncontrolled periodontal disease; (2) inability to provide sufficient connective tissue; (3) maxillofacial deformity, cysts, or tumors; (4) defective dentition, supernumerary teeth, impacted teeth, and/or abnormal tooth morphology; (5) caries, residual roots, or apical periodontitis; (6) medication that could affect bone metabolism; and (7) tooth rotation and severe dental crowding.

Based on previous study and preexperiment, the study outcome variable was set to be the horizontal labial bone thickness changes around mandibular canine teeth at 6 mm below the CEJ during treatment [16, 17]. PASS software 11.0 was used to calculate the required sample size, with the results showing that a sample size of 10 achieves 83% power to detect a mean of paired differences of 0.32 with an estimated standard deviation of differences of 0.3 and with a significance level (alpha) of 0.05 using a two-sided paired t-test. So we included 60 teeth with 11 Center incisors, 11 Lateral incisors, 19 Canines and 19 Premolars.

All participants were informed about the treatment plan and possible periodontal complications, and written consent was obtained. The final sample of 60 mandibular teeth was selected according to the inclusion and exclusion criteria (Supplementary Table S1) and was divided into four groups according to tooth position: central incisors, lateral incisors, canines, and premolars. Participants were enrolled after ensuring the absence of systematic diseases and contraindications, and a strict protection protocol was followed during CBCT.

### Surgical procedure

Partial-thickness flaps were reflected after sulcular and vertical-release incisions around the labial surface. Preserved gingival papillae are beneficial for achieving the maximum blood supply and reducing postoperative scar contractions. Partial-thickness connective tissue grafts (1.5 mm) matching the grafting site were harvested from the palate and sutured on the recipient beds. The covering flaps were repositioned coronally and sutured without tension. Patients were prescribed anti-inflammatory drugs for 3 days, and sutures were removed 1–2 weeks after surgery. All periodontal surgeries were performed by the same experienced periodontist.

### Radiographic measurement

To assess the alveolar bone conditions in patients with thin gingival biotypes, CBCT was performed twice: before treatment (T0) and at least 6 months after grafting surgery (T1). Scanning was performed with patients in an upright position with maximum intercuspation. CBCT images were acquired using a Planmeca Romexis CBCT scanner (Planmeca Romexis Viewer 3.8.3.R) with 360° rotation, scan time 12 s, and field of view 501 × 501 × 466 mm^3^ at 96 kV and 10 mA, with a voxel size of 200. The data were converted to DICOM format, imported into the Dolphin 3D Imaging software (Dolphin Imaging and Management Solution, Chatsworth, CA), and reconstructed with a slice thickness of 0.5 mm for analysis.

**Fig. 1:**
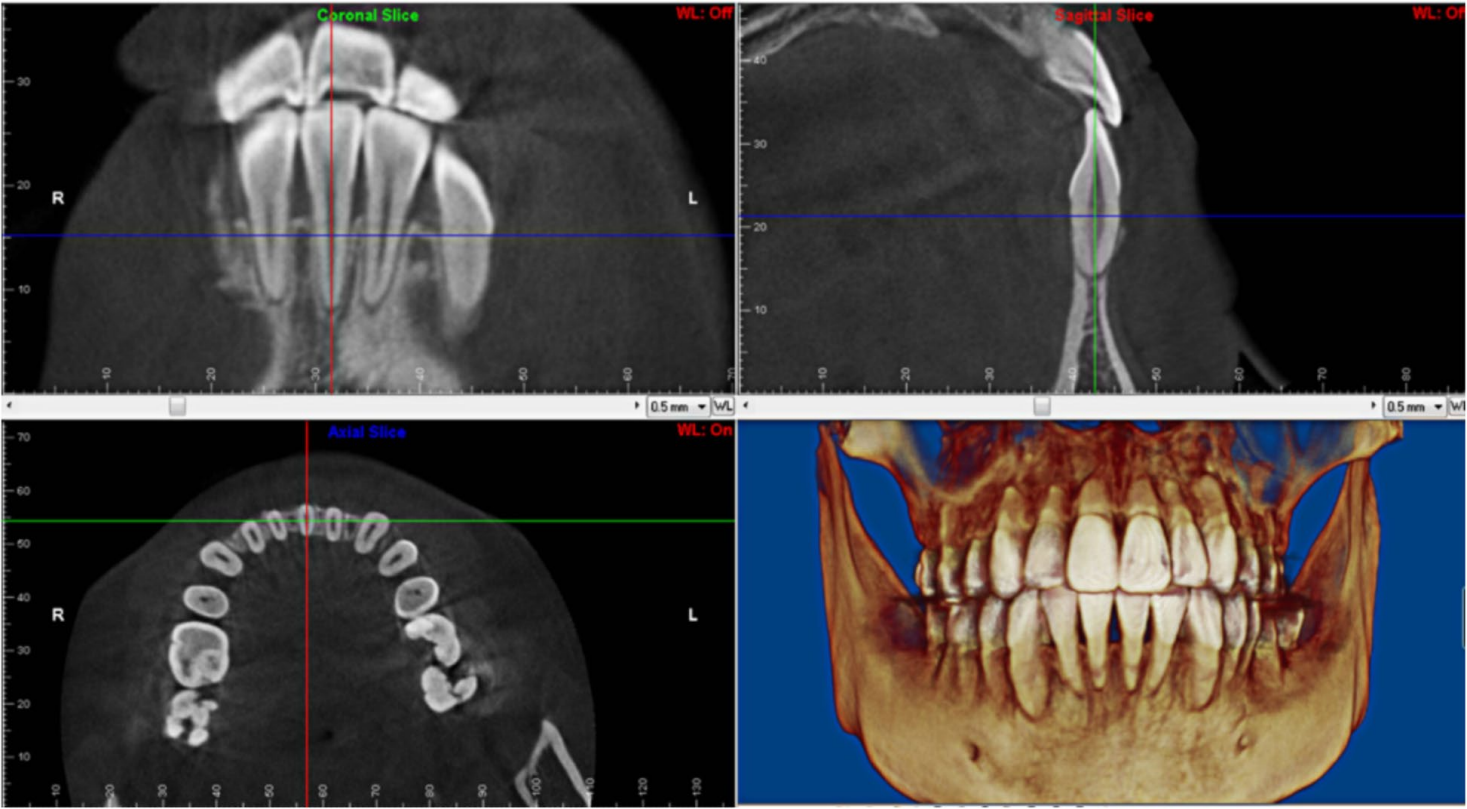
Detailed procedure for locating. Blue lines represent the axial plane, red lines represent the sagittal plane, and green lines represent the coronal plane. These reference lines were adjusted through the axis of the tooth in the three views

**Fig. 2:**
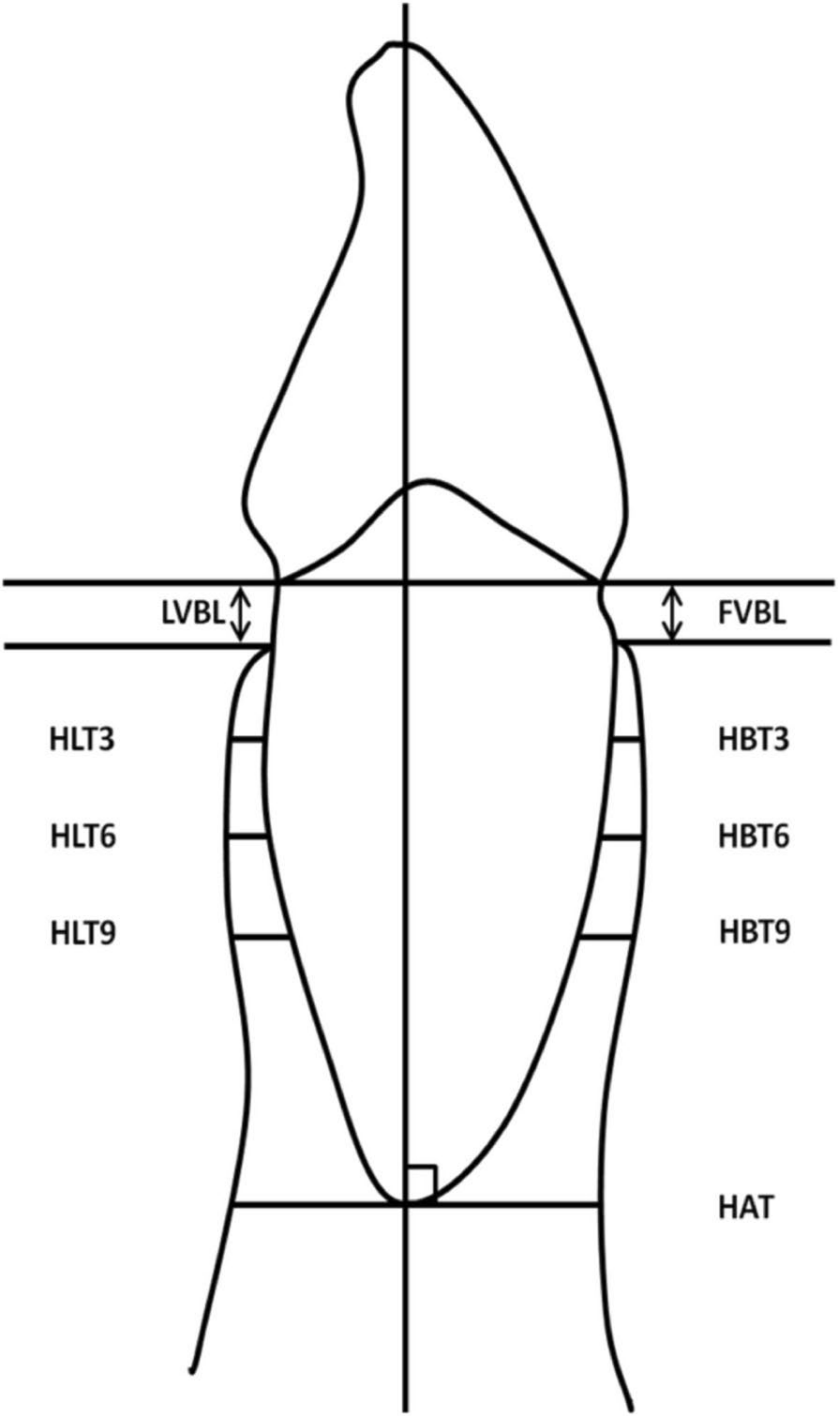
Landmarks and measurements. FVBL: facial vertical bone level, LVBL: lingual vertical bone level, HBT: horizontal labial bone thickness

**Fig. 3:**
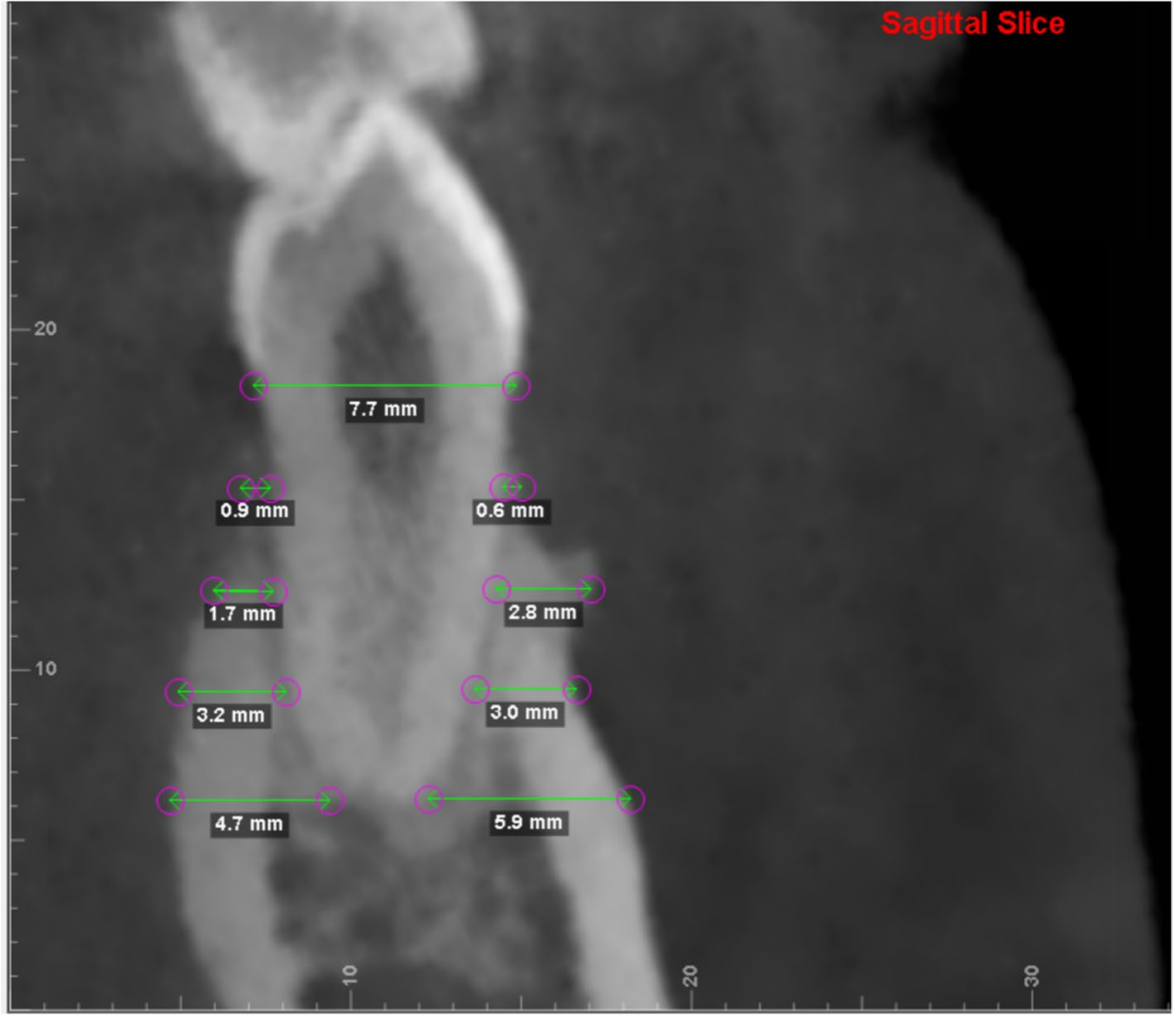
Measurements on CBCT scans

Measurements primarily included horizontal labial alveolar bone width and vertical bone level at T0 and T1. In the sagittal plane, the horizontal labial bone thickness was measured in a buccolingual direction perpendicular to the long axis of each tooth (Fig. 1) at 3, 6, and 9 mm from the CEJ and at the root apex [16]. In addition, the vertical bone level was measured parallel to the root axis from the CEJ to the alveolar crest on the facial and lingual aspects, as described by Ahn et al. [17] (Figs. 2 and 3).

### Statistical analysis

All measurements were repeated twice over the course of 2 weeks by the same observer. The interclass correlation coefficient was calculated to assess systemic intra-examiner errors between the two measurements. As there were no significant differences between the two measurements, all teeth were included, and the mean of the two values was calculated for each tooth. All statistical analyses were performed using SPSS version 25 (IBM Corporation, Armonk, NY, USA), and statistical significance was set at *P* < 0.05.

The Shapiro–Wilk test was used to test the normality between the measurements at T0 and T1. An analysis of variance was performed to assess changes according to the tooth position. A paired t-test was used to reveal a normal distribution of the measures; for non-normally distributed variables, the Wilcoxon signed-rank test was used for comparisons.

## Results

### Clinical observation

**Fig. 4:**
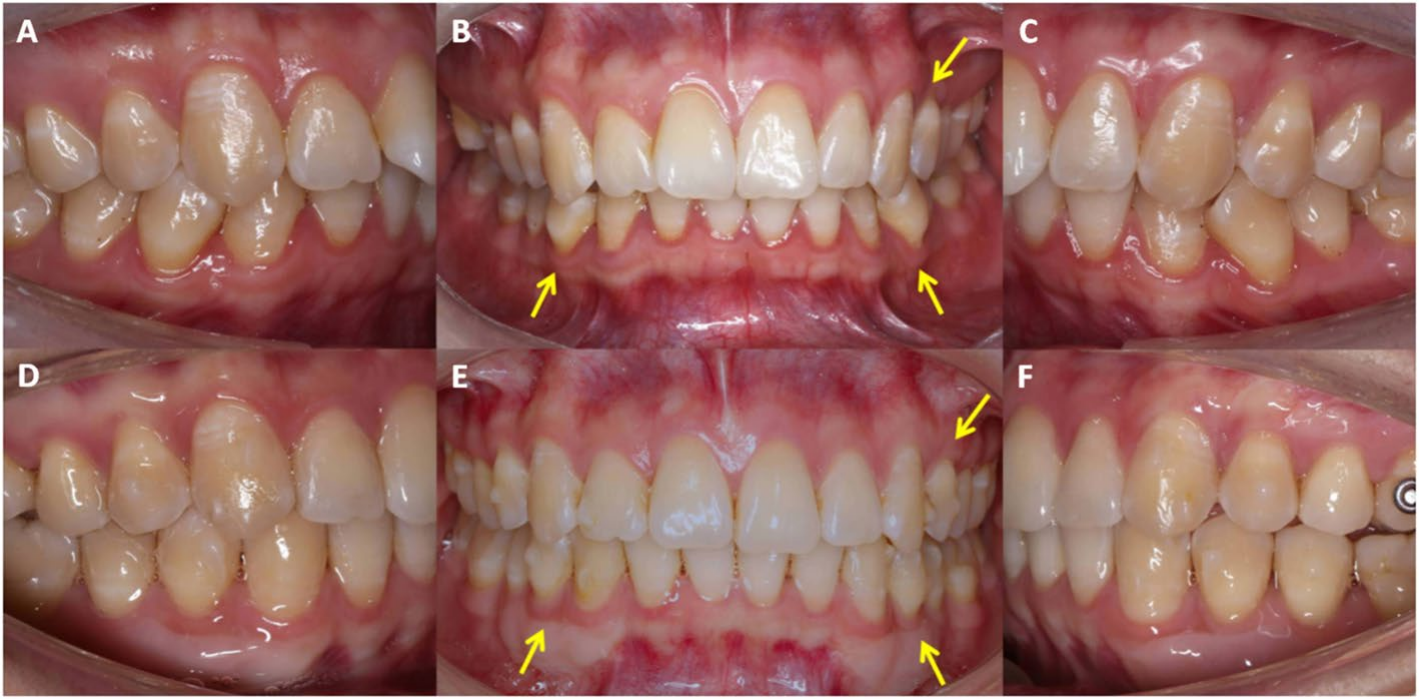
**A**, **B**, **C** Mucogingival condition before surgery. The maxillary left premolar and mandibular canines have gingival recessions, and the orthodontic treatment requires arch expansion for alignment. Autogenous soft tissue grafting has been performed in maxillary left premolar and mandibular canine and premolars. **D**, **E**, **F**: Changes 2 years after surgery. No further recession is observed in the operated regions after orthodontic treatment completion

After surgery, patients were followed up for at least 6 months. Compared to T0, the thickness and width of keratinized gingiva increased at T1, and no further recession nor bone dehiscence or fenestration occurred after orthodontic proclination or labial movement of teeth (Fig. 4). Two cases of typical thin gingival biotypes who underwent orthodontic treatment with autogenous soft tissue grafting at our institution were presented in Supplementary Fig. 1–4.

### Radiographic evaluation

From T0 to T1, both horizontal labial bone thickness and vertical bone height showed a minimal increase in patients who underwent autogenous soft tissue grafting surgery.

### Horizontal labial bone thickness

**Fig. 5:**
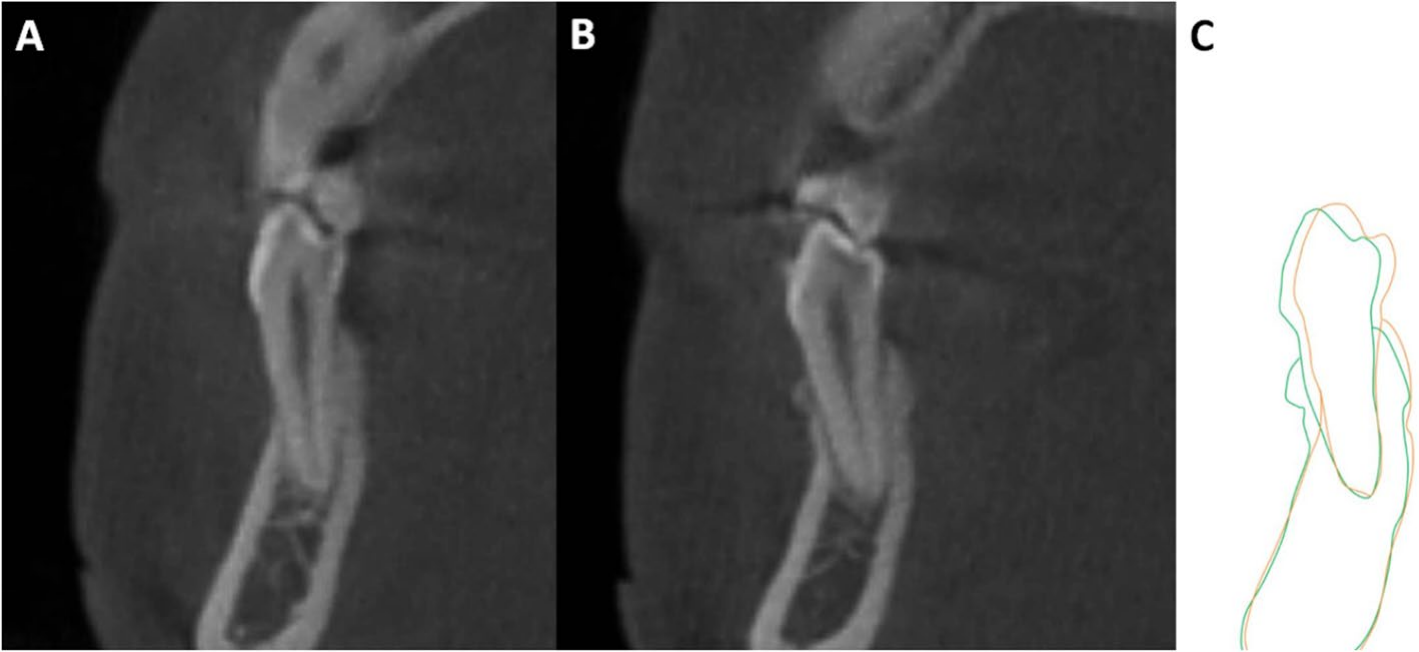
Superimposition of the two images before and after periodontal surgery. White represents T0; green represents T1. The tooth has been proclined with new labial bone formation

CBCT showed a minimal increase in the thickness of the labial bone after periodontal surgery (Fig. 5), and preoperative bony defects also improved. Table 1 shows the changes in horizontal labial bone thickness from T0 to T1. The values of almost all measurements increased. For the central incisors, a slight increase was observed in the total bone area, especially at 6 mm below the CEJ, and the changes were statistically significant (*P* = 0.036). For the lateral incisors, the labial thickness increased in the total bone area, and the differences at 6 mm below the CEJ were statistically significant (*P* < 0.05). The values of all measurements increased in the canine group, and the differences were statistically significant (*P* < 0.05). The labial bone thickness in the premolars increased by 0.04 mm at the apical level, but the increase was not statistically significant (*P* = 0.804). The labial bone was thicker in other areas, and the changes were statistically significant (*P* < 0.05).

**Table 1:** Horizontal labial bone thickness changes around mandibular teeth during treatment

Measurements	T0 stage		T1 stage		T1-T0		
	Mean	SD	Mean	SD	Mean	SD	*P* value*
Central incisor							
HBT3(mm)	0.43	0.45	0.45	0.31	0.02	0.37	0.918
HBT6(mm)	0.32	0.33	0.60	0.63	0.28	0.44	0.036*
HBT9(mm)	1.09	1.20	1.25	1.36	0.15	0.57	0.331
HBTA(mm)	7.74	3.23	7.86	4.12	0.13	1.33	0.553
Lateral incisor							
HBT3(mm)	0.44	0.37	0.56	0.40	0.13	0.26	0.137
HBT6(mm)	0.17	0.13	0.75	0.96	0.57	0.92	0.014*
HBT9(mm)	0.59	0.64	1.13	1.69	0.55	1.19	0.068
HBTA(mm)	7.41	3.44	7.93	5.14	0.51	2.28	0.514
Canine							
HBT3(mm)	0.38	0.35	0.81	0.66	0.43	0.65	0.007**
HBT6(mm)	0.22	0.27	0.54	0.31	0.32	0.24	0.000**
HBT9(mm)	0.55	0.56	0.88	0.61	0.33	0.45	0.004**
HBTA(mm)	9.24	2.16	9.71	2.82	0.47	1.02	0.049*
Premolar							
HBT3(mm)	0.49	0.24	0.68	0.28	0.19	0.24	0.005**
HBT6(mm)	0.67	0.43	0.97	0.37	0.30	0.39	0.005**
HBT9(mm)	1.43	0.65	1.77	0.65	0.34	0.57	0.027*
HBTA(mm)	11.25	2.06	11.29	2.10	0.04	0.67	0.804

*HBT* horizontal bone thickness, *HBTA* horizontal bone thickness at the apex level

T0, before treatment; T1, at least six months after surgery

^*^P < 0.05; **P < 0.01

### Vertical bone level

Table 2 shows that the vertical bone levels on the facial and lingual aspects exhibited minimal decrease in all groups. The facial vertical bone levels decreased, but the changes were not statistically significant. The central and lateral incisors showed vertical bone gain on the lingual aspect, and the differences were statistically significant (*P* < 0.05). Vertical bone levels decreased in the canine and premolar regions, but the changes were not statistically significant.

**Table 2:** Vertical bone level around the mandibular teeth during treatment

Measurements	T0 stage		T1 stage		T1-T0		
	Mean	SD	Mean	SD	Mean	SD	*P* value*
Central incisor							
FVBL(mm)	2.98	2.09	2.35	2.07	-0.64	2.43	0.241
LVBL(mm)	2.23	1.44	1.43	0.43	-0.80	1.57	0.016*
Lateral incisor							
FVBL(mm)	2.25	1.60	2.16	1.30	-0.09	1.16	0.574
LVBL(mm)	2.35	1.24	1.83	0.95	-0.51	0.59	0.016*
Canine							
FVBL(mm)	3.03	2.70	1.83	0.86	-1.20	2.35	0.059
LVBL(mm)	1.19	1.39	0.88	0.61	-0.31	1.27	0.078
Premolar							
FVBL(mm)	2.02	1.45	1.62	0.78	-0.29	0.89	0.363
LVBL(mm)	1.32	0.58	1.22	0.45	-1.00	0.56	0.472

*FVBL* facial/labial vertical bone level, *LVBL* lingual vertical bone level

T0, before treatment; T1, post-surgical at least six months

^*^P < 0.05; ^**^P < 0.01

Supplementary Table S1 shows the comparison of alveolar bone changes according to tooth positions, and no statistically significant differences were noted.

## Discussion

Orthodontic treatment is characterized by alveolar bone remodeling. Osteoclasts located on the inner surface of the bone plate in the direction of tooth movement are activated to induce bone resorption, whereas osteoblasts located outside the plate proliferate to form new bone. Therefore, with correct orthodontic force, bone thickness can be maintained without bone defects. As the number of adult patients undergoing orthodontic treatment is increasing, the incidence of bone dehiscence and fenestration before [18] and during orthodontic movements may also increase due to slow bone metabolism and poor periodontal conditions, like thin scalloped gingival biotypes.

Previous studies have shown that patients with a thin alveolar bone and narrow attached gingiva, especially those with poor oral hygiene, are more likely to experience gingival recession during orthodontic treatment [19]. Thin alveolar bone or bone dehiscence makes the periodontal tissue highly susceptible to bacterial plaque-induced inflammation [20, 21], and inflammatory changes can block osteogenesis and aggravate bone defects. Narrow keratinized gingiva or thin gingival biotypes cannot effectively resist mechanical force or dental plaque invasion [22], and attachment loss and marginal recession can easily develop, leading to repositioning of the roots outside the cortical bone.

Autogenous tissue grafting has been proposed in previous studies to prevent periodontal loss and have obtained expected clinical results. Most researchers attribute these effects to the increase in the amount of keratinized tissue, which is thought to be a physical barrier to isolated plaque invasion [23]. Indeed, in this study, we also observed gingival augmentation and no further recession after proclination or labial movement of teeth, which is in consistent with previous studies. However, the ideal goal of periodontal regeneration should include not only new connective tissue attachment, but also bone formation. Thus, morphological changes in the alveolar bone were further measured using CBCT. It is worth mentioning that new bone regeneration in the direction of orthodontic movement was observed after autogenous soft tissue grafting, which may prevent periodontal recession during labial movement of teeth and ensure long-term stability of teeth.

This study aimed to provide periodontal protection during orthodontic proclination or labial movement of teeth in patients with a thin gingival biotype. To evaluate and predict the periodontal risk and surgical prognosis, the morphological changes in the alveolar bone before and after grafting should be measured and compared. Previous clinical observations of the alveolar bone were based on two-dimensional radiographs or histometric evaluations. Conventional radiographs may exhibit significant overlap, and it is difficult to accurately assess the position and bony support of the root. Histological measurements are difficult to obtain, due to surgical trauma and ethical considerations. Misch et al. showed no significant differences between CBCT and the gold standard histological measurements [24], and indicated that CBCT can help evaluate bone defects in three dimensions accurately and reliably. Thus, CBCT images were used in this study to assess the changes in bone height and width.

CBCT showed a minimal increase in the thickness of the labial bone after periodontal surgery, and preoperative bony defects also improved. The labial bone thickness at 3 mm and 6 mm below the CEJ was < 0.5 mm before surgery for most tooth but increased to > 0.5 mm after surgery. The vertical bone level was determined using the positions of the alveolar crest and CEJ. The distance from the CEJ to the bone crest, representing the biological width of supracrestal connective tissue attachments, was between 1.5 and 2.5 mm for most tooth and was generally < 4 mm [25, 26]. In this study, the vertical bone levels at T0 were > 2 mm for most tooth. Especially in the central incisors and canines, they were > 2.5 mm on the labial side, indicating a pretreatment bone loss. After surgery, the vertical bone height increased, and a small amount of bone regeneration was observed at the alveolar crest. Although the differences were not statistically significant, bone heights were normal. These imaging changes confirmed new bone formation and osteogenesis, similar to the minimal bone gain observed after connective tissue grafting in several case reports [6, 8, 9, 27] and animal studies [28, 29].

This study confirmed the widening of keratinized tissue and new bone formation. However, there were no significant differences in new bone formation in different tooth. According to our findings, tooth positions may not lead to a statistically significant difference in bony changes. The mechanism of osteogenesis remains unclear, but previous studies have described the following possible mechanisms: (1) Potential periosteal traumatic injury occurs during surgery. Bony exostoses were found following soft tissue grafting in some cases, and dense, mature lamellar bone surrounded by fibrous tissue was observed histologically. It has been speculated that surgical injury and periosteal fenestration may be responsible for bony exostoses [26]. Some authors have suggested that osteoprecursor cells in the connective tissue may be activated by grafting procedures, resulting in osteogenesis. [30] (2) Multiple case reports have reported newly formed bone following connective tissue grafting on the root surface [8–10]. The authors indicated that a thick gingival graft can be used as a biological membrane to create space between the root surface and gingiva. This membrane prevents epithelial cell proliferation and early contact, allowing the periodontal ligament cells to repopulate coronally [6–8]. However, another study found no evidence of new bone development after open flap procedures, and reported bone loss [31]. Harris et al. suggested that deeper recession caused epithelial cells to cover longer distances and that stem cells could migrate and induce periodontal regeneration [7, 32]. (3) Orthodontic tooth movement is accompanied by bone remodeling, but thin gingival tissue or bony defects may inhibit bone regeneration and promote bone loss due to susceptibility to bacteria [20, 33]. Augmented keratinized gingiva is a physical barrier to isolated plaque invasion and provides a stronger environment that contributes to bone remodeling. (4) Surgery creates a complex environment that provides an adequate blood supply and transports growth factors into the recipient bed, all of which may induce differentiation of pluripotent or mesenchymal cells in the periosteum or periodontal ligament into osteoblasts and promote bone generation [34]. Indeed, the gingiva is a rich source of mesenchymal stem cells, which can support sufficient cell differentiation [35, 36].

Despite all the advantages, our study still had some limitations. Firstly, CBCT has limitations in evaluating bone thickness due to spatial resolution, partial volume averaging, etc.. Secondly, further evaluation is needed to analyse the long-term stability of the technique. Thus, clinical studies with larger samples and long-term data are needed to validate its use. Last but not least, there are many speculations about the mechanism of osteogenesis, and additional clinical trials and histologic analyses are required to verify osteogenesis and investigate the possible mechanisms in the future.

## Conclusions

New bone regeneration in the direction of orthodontic movement was observed on CBCT images after autogenous soft tissue grafting. The new bone and augmented keratinized gingiva may prevent periodontal recession during proclination or labial movement of teeth and ensure long-term stability of oral health. If the feasibility and underlying mechanism of this technique are elucidated in future studies, it can be used to develop a new treatment for bone recession in periodontal disease.

## Supplementary Information

**Additional file 1.**

## Data Availability

The data of the fndings in this study are available from the corresponding author upon reasonable request.

## Abbreviation

CBCT: Cone beam computed tomography
CEJ: Cementoenamel junction
FVBL: Facial vertical bone level
LVBL: Lingual vertical bone level
HBT: Horizontal labial bone thickness
