# Supplementary material for: Morphological changes in alveolar bone thickness and height after orthodontic proclination or labial movement combined with autogenous soft tissue grafting: a CBCT evaluation
Source: BMC Oral Health. 2023 Apr 15;23:218. doi: 10.1186/s12903-023-02944-w (PMC10105956; doi:10.1186/s12903-023-02944-w)
Supplement: Supplementary file 1 — Additional file 1. [file 12903_2023_2944_MOESM1_ESM.docx]

**Morphological changes in alveolar bone thickness and height after orthodontic proclination or labial movement combined with autogenous soft tissue grafting: a CBCT evaluation.**

Tianyu Zhang^1,+^ , Lingling Zhang^1,+^, Min Li^2^, Fang Yi^1^, Chengri Li^1^, Yanqin Lu^1,*^

^1^ Department of Orthodontics, Hunan Key Laboratory of Oral Health Research & Hunan Clinical Research Center of Oral Major Diseases and Oral Health & Xiangya Stomatological Hospital & Xiangya School of Stomatology, Central South University，Changsha, 410008, Hunan, China.

^2^ Department of Periodontics, Hunan Key Laboratory of Oral Health Research & Hunan Clinical Research Center of Oral Major Diseases and Oral Health & Xiangya Stomatological Hospital & Xiangya School of Stomatology, Central South University，Changsha, 410008, Hunan, China.

^+^ Tianyu Zhang and Lingling Zhang contributed equally to this study.

Corresponding Author: Y. Lu, Professor, PhD., Email: [213031@csu.edu.cn](mailto:213031@csu.edu.cn)

^+^ Tianyu Zhang and Lingling Zhang contributed equally to this study.

Corresponding Author: Y. Lu, Professor, PhD.,

Department of Orthodontics, Hunan Key Laboratory of Oral Health Research & Hunan Clinical Research Center of Oral Major Diseases and Oral Health & Xiangya Stomatological Hospital & Xiangya School of Stomatology, Central South University，Changsha, 410008, Hunan, China.

Telephone：+86 13973102658

Email: [213031@csu.edu.cn](mailto:213031@csu.edu.cn)

Author information:

Tianyu Zhang, Email: [ilonew@163.com](mailto:ilonew@163.com)

Lingling Zhang, Email: [zhanglingling@whu.edu.cn](mailto:zhanglingling@whu.edu.cn)

Min Li, Email: [liminlaxin@126.com](mailto:liminlaxin@126.com)

Fang Yi, Email: [yifangcsu@163.com](mailto:yifangcsu@163.com)

Chengri Li, Email: [lcr0928@hotmail.com](mailto:lcr0928@hotmail.com)

Yanqin Lu, [213031@csu.edu.cn](mailto:213031@csu.edu.cn)

***Supplementary Table S1.*** Patient basic characteristic.

| **Variable** | **N** | **%** |
| --- | --- | --- |
| Age | Mean 25.26±7.24 years | |
| 19-20 | 5 | 31.25% |
| 20-29 | 9 | 56.25% |
| 30-39 | 1 | 6.25% |
| 40 and over | 1 | 6.25% |
| Gender |  | |
| Male | 4 | 25.00% |
| Female | 12 | 75.00% |
| Tooth position |  | |
| Center incisor | 11 | 18.33% |
| Lateral incisor | 11 | 18.33% |
| Canine | 19 | 31.67% |
| Premolar | 19 | 31.67% |


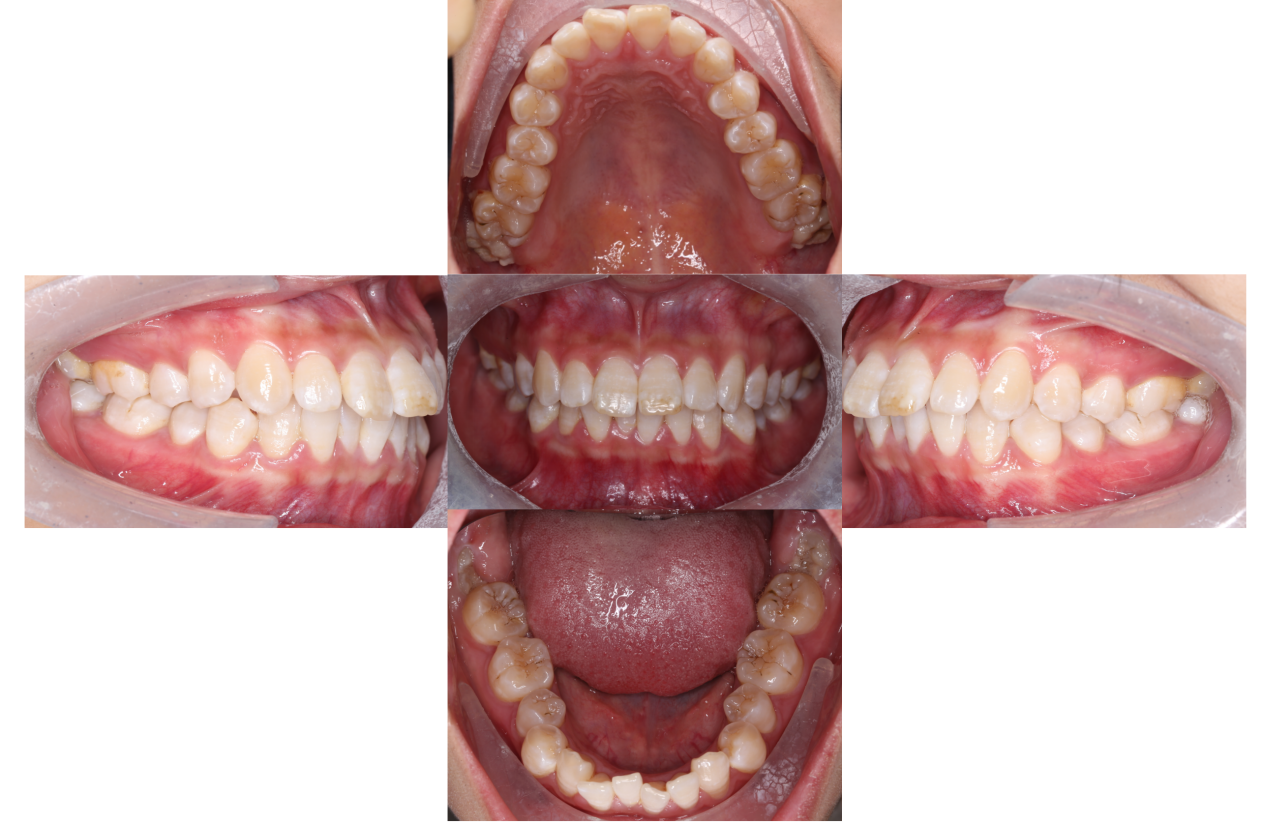


*Supplementary Figure 1.*  Data of patient A before orthodontic treatment.


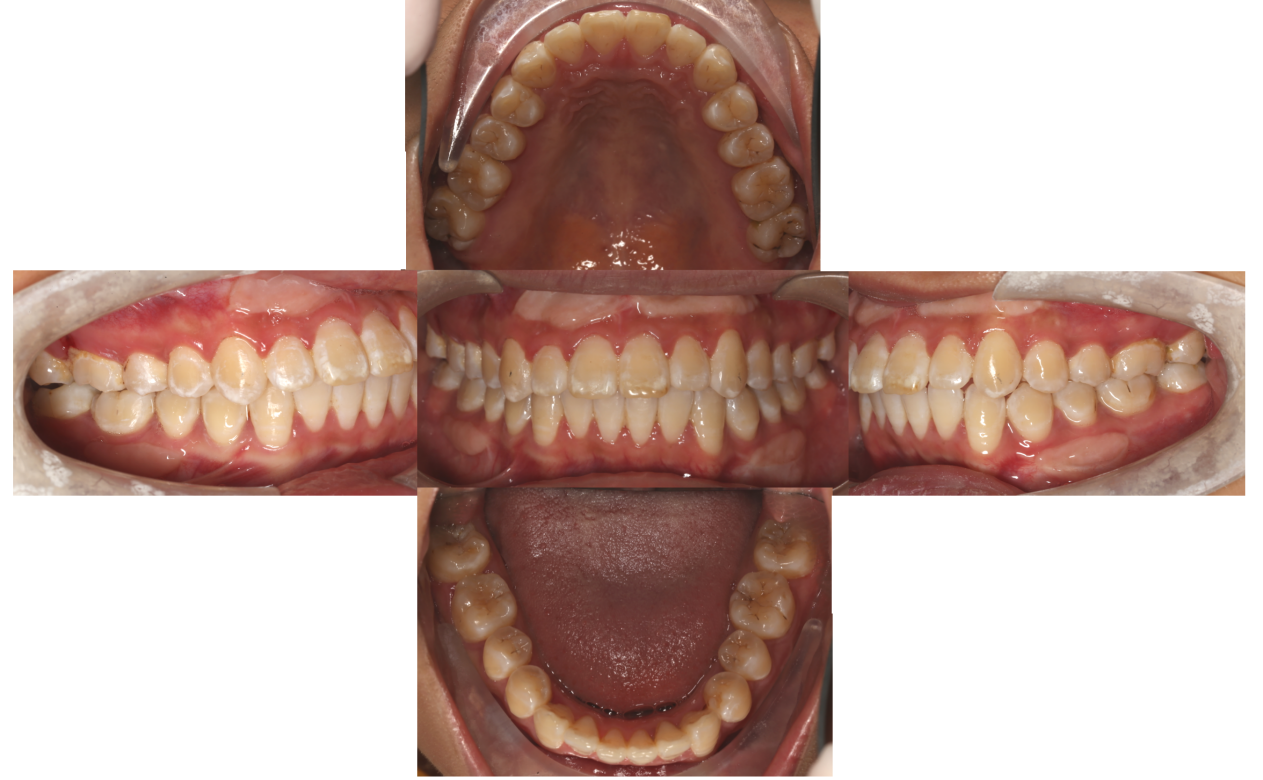


*Supplementary Figure 2.*  Data of patient A after orthodontic treatment.


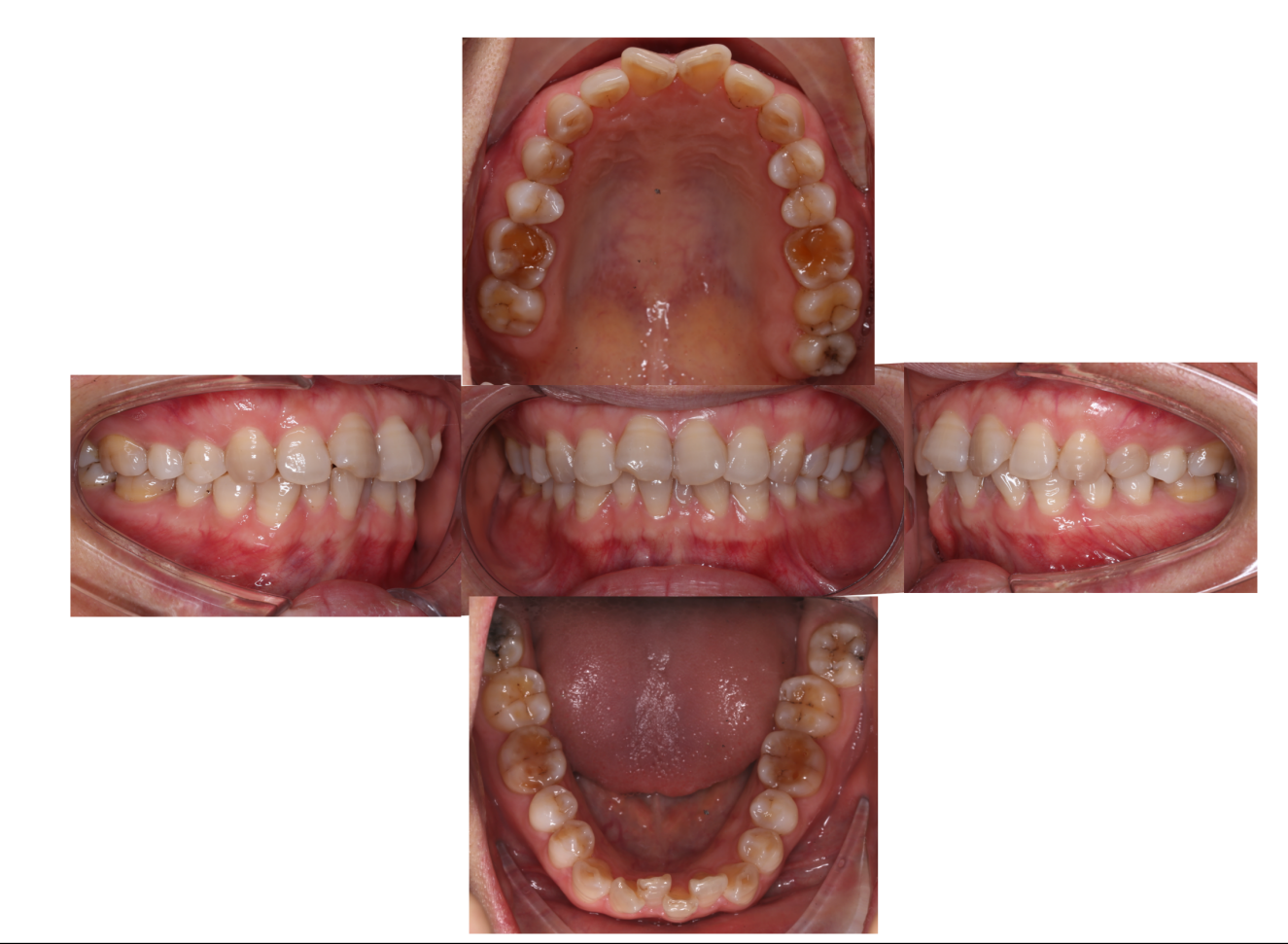


*Supplementary Figure 3.*  Data of patient B before orthodontic treatment.


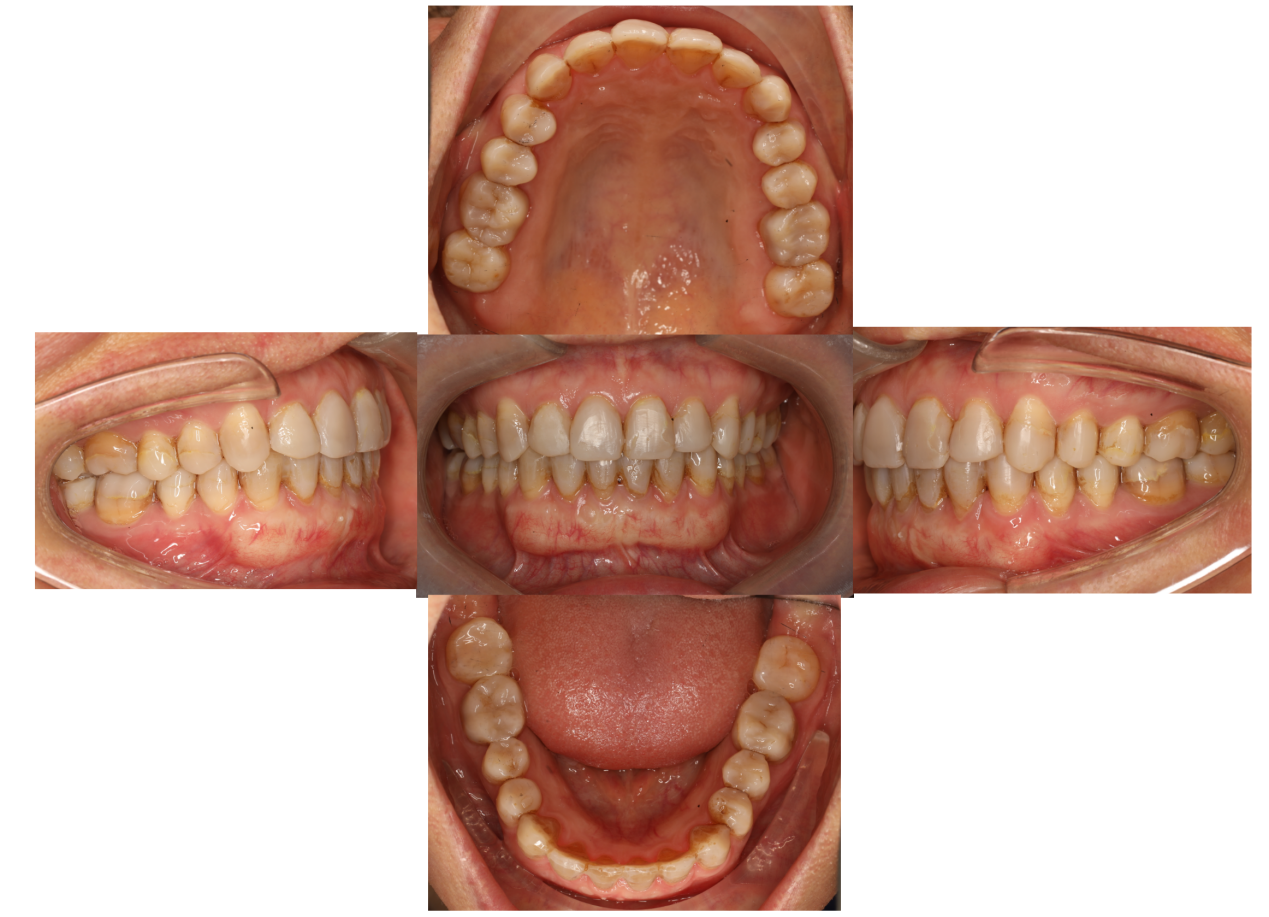


*Supplementary Figure 4.*  Data of patient B after orthodontic treatment.
